# Correction: Double triage to identify poorly annotated genes in maize: The missing link in community curation

**DOI:** 10.1371/journal.pone.0314495

**Published:** 2024-11-21

**Authors:** Marcela K. Tello-Ruiz, Cristina F. Marco, Fei-Man Hsu, Rajdeep S. Khangura, Pengfei Qiao, Sirjan Sapkota, Michelle C. Stitzer, Rachael Wasikowski, Hao Wu, Junpeng Zhan, Kapeel Chougule, Lindsay M. Barone, Cornel Ghiban, Demitri Muna, Andrew C. Olson, Liya Wang, Doreen Ware, David A. Micklos

The 12^th^ author’s middle initial is incorrect. The correct name is: Lindsay M. Barone.
