# Supplementary material for: Double triage to identify poorly annotated genes in maize: The missing link in community curation
Source: PLoS One. 2019 Oct 28;14(10):e0224086. doi: 10.1371/journal.pone.0224086 (PMC6816542; doi:10.1371/journal.pone.0224086)
Supplement: S1 Appendix — (DOCX) [file pone.0224086.s001.docx]

**S1 Appendix. Survey Results**

**Table 1: Inbred lines used in survey respondent research**

|  | Count | % |
| --- | --- | --- |
| B73 | 98 | 87.50% |
| W22 | 64 | 57.14% |
| NC350 | 23 | 20.54% |
| Teosinte inbred (TIL11) | 22 | 19.64% |
| None of the above | 11 | 9.82% |

*n = 112, participants could select more than one option*

**Table 2: Tissues used in survey respondent research**

|  | Count | % |
| --- | --- | --- |
| Immature ears | 48 | 43.24% |
| Root tips | 44 | 39.64% |
| Roots | 43 | 38.74% |
| Shoot tips | 40 | 36.04% |
| Mature pollen | 40 | 36.04% |
| Embryo 15 dap | 38 | 34.23% |
| Endosperm 15 dap | 36 | 32.43% |
| FACS-sorted cell types | 22 | 19.82% |
| None of the above | 24 | 21.62% |

*n = 111, participants could select more than one option*

**Table 3: Data sets important to respondent research**

|  | Count | % |
| --- | --- | --- |
| RNA-seq | 95 | 84.82% |
| Whole genome sequences | 89 | 79.46% |
| Transcription factor binding sites | 66 | 58.93% |
| Open chromatin marks | 51 | 45.54% |
| DNA methylation sites | 51 | 45.54% |
| Small RNA-seq | 47 | 41.96% |
| Histone modifications | 43 | 38.39% |
| RNA polymerase II binding sites | 32 | 28.57% |
| None of the above | 4 | 3.57% |

*n = 112, participants could select more than one option*

**Table 4: Types of anticipated analyses respondents would do with students**

|  | Individual Projects | | Class Projects | |
| --- | --- | --- | --- | --- |
|  | Count | % | Count | % |
| Compare regulatory elements of different inbred lines | 58 | 87.88% | 17 | 25.76% |
| Compare genome structure of different inbred lines | 70 | 87.50% | 21 | 26.25% |
| Compare members of specific gene families | 67 | 85.90% | 27 | 34.62% |
| Compare transposons of different inbred lines | 43 | 82.69% | 14 | 26.92% |

*n = 95, participants could select more than one option.*

**Table 5: Utility of hand-edited maize gene family annotations for research**

|  | Count | % |
| --- | --- | --- |
| Not useful | 2 | 1.82% |
| Slightly useful | 9 | 8.18% |
| Useful | 34 | 30.91% |
| Moderately useful | 23 | 20.91% |
| Extremely useful | 42 | 38.18% |

**Table 6: Willingness of respondents to participate in specific activities with training**

|  | Count | % |
| --- | --- | --- |
| Evaluate community annotations of a gene family of which you have expert knowledge | 63 | 61.17% |
| Participate in annotating a gene family of interest | 63 | 60.00% |
| Annotate a gene family as a class project | 41 | 41.41% |

**Table 7: Overall bioinformatics skill level**

|  | Count | % |
| --- | --- | --- |
| Never used bioinformatics tools | 7 | 6.31% |
| Beginner | 38 | 34.23% |
| Intermediate | 42 | 37.84% |
| Advanced | 24 | 21.62% |

**Table 8: Respondents working with Big Data**

|  | Count | % |
| --- | --- | --- |
| Yes, I currently work with Big Data | 85 | 76.58% |
| No, I do not currently work with Big Data | 26 | 23.42% |

**Table 9: Respondents working with Big Data in the next three years**

|  | Count | % |
| --- | --- | --- |
| Yes, I will | 100 | 90.09% |
| No, I won’t | 11 | 9.91% |

**Table 10: Types of maize research done by survey respondents**

|  | Count | % |
| --- | --- | --- |
| Computational and Large-scale Biology | 47 | 43.52% |
| Quantitative Genetics and/or Breeding | 41 | 37.96% |
| Biochemical and Molecular Genetics | 39 | 36.11% |
| Cell and/or Developmental Biology | 36 | 33.33% |
| Population Genetics/Genomics | 30 | 27.78% |
| Transposons and Epigenetics | 16 | 14.81% |
| Cytogenetics | 11 | 10.19% |
| Education and Outreach | 8 | 7.41% |
| Other | 3 | 2.78% |

*n = 108, participants could select more than one option*

**Table 11: Positions held by survey respondents**

|  | Count | % |
| --- | --- | --- |
| Researcher (Independent or PI) | 41 | 37.96% |
| Graduate Student | 28 | 25.93% |
| Post-doc | 19 | 17.59% |
| Faculty/Educator | 11 | 10.19% |
| Undergraduate Student | 10 | 9.26% |
| Industry | 5 | 4.63% |
| Other (please specify) | 4 | 3.70% |

*n = 108, participants could select more than one option.*
