# Supplementary material for: Double triage to identify poorly annotated genes in maize: The missing link in community curation
Source: PLoS One. 2019 Oct 28;14(10):e0224086. doi: 10.1371/journal.pone.0224086 (PMC6816542; doi:10.1371/journal.pone.0224086)
Supplement: S1 Table — (DOCX) [file pone.0224086.s002.docx]

**S1 Table. Annotation errors and MAKER-P quality metrics for targeted families**

| **Gene ID** | **Gene name** | **Transcript count** | **Canonical transcript** | **Quality -flagged transcript** | **AED** | **QI2** | **Missing exons** | **Extra exons** | **Different exon length/composition** | **Evidence accounted by other transcripts** | **Different UTR length** |
| --- | --- | --- | --- | --- | --- | --- | --- | --- | --- | --- | --- |
| Zm00001d043179 | PIN9 | 1 |  |  | 0.01 | 0.5 |  |  | X |  |  |
| Zm00001d006082 | PIN11 | 1 |  |  | 0.12 | 0.3 | X |  |  |  |  |
| Zm00001d007395 | ZmGH3-3 | 1 |  |  | 0.02 | 0.5 | X |  |  |  |  |
| Zm00001d011377 | JAR-1 | 3 | T1 | T1 | 0.1 | 0.6 |  |  | X (T1) | T2 is correct | X (T1/T3) |
| Zm00001d029577 | ZmGH3-1 | 3 | T2 | T2 | 0.06 | 0.5 | X (T1) |  | X (T1/T2) | T3 is correct |  |
| Zm00001d051514 | ABCB1 | 4 | T2 | T4 | 0.42 | 0.66 | X (T1) |  | X (T1/T3) | T2 is correct | X (T1) |
| Zm00001d050259 | ABCA1 | 3 | T2 | T1 | 0.42 | 0.5 | X (T4) |  | X (T2) | T3 is correct |  |
| Zm00001d041947 | ABCB21 | 1 |  |  | 0.3 | 0.7 |  |  | X |  | X |
| Zm00001d031871 | ABCB12 | 2 | T1 | T1 | 0.01 | 0.75 |  |  | X (T1) | T2 is correct |  |
| Zm00001d034087 | ORC-5 | 1 |  |  | 0.22 | 0.6 | X | X | X |  |  |
| Zm00001d039371 | TCP-33 | 3 | T1 | T1 | 0.12 | 0.5 |  | X (T1) | X (T1) | T2 & T3 are correct |  |
