# Supplementary material for: Double triage to identify poorly annotated genes in maize: The missing link in community curation
Source: PLoS One. 2019 Oct 28;14(10):e0224086. doi: 10.1371/journal.pone.0224086 (PMC6816542; doi:10.1371/journal.pone.0224086)
Supplement: S2 Table — (DOCX) [file pone.0224086.s003.docx]

**S2 Table. Classical genes curated in Apollo**

| **Gene ID from Gramene** | **Error detection method** | **Transcript count** | **Canonical transcript** | **Quality-flagged transcript** | **AED** | **QI2** | **Missing exons** | **Extra exons** | **Non-canonical splice site** | **Different exon length/**  **composition** | **Evidence accounted by other transcripts (includes a mix of transcripts)** | **Different UTR length** |
| --- | --- | --- | --- | --- | --- | --- | --- | --- | --- | --- | --- | --- |
| Zm00001d026088 | Gene Tree & AED/QI2 | 2 | T2 | T1 | 0.09 | 0.71 | X |  |  | X | X |  |
| Zm00001d003533 | Gene Tree & AED/QI2 | 2 | T1 | T1 | 0.12 | 0.66 |  |  | X | X | X |  |
| Zm00001d045563 | Gene Tree & AED/QI2 | 2 | T1 | T1 | 0.13 | 0.6 | X |  | X | X | X |  |
| Zm00001d048082 | Gene Tree & AED/QI2 | 4 | T1 | T1 | 0.14 | 0.71 |  |  |  | X | X | X |
| Zm00001d031620 | Gene Tree & AED/QI2 | 2 | T1 | T1 | 0.15 | 0.75 |  | X |  | X | X | X |
| Zm00001d029074 | Gene Tree & AED/QI2 | 3 | T1 | T1 | 0.17 | 0.66 |  | X | X | X | X |  |
| Zm00001d008882 | Gene Tree & AED/QI2 | 2 | T1 | T1 | 0.26 | 0.62 |  |  |  | X |  |  |
| Zm00001d053899 | Gene Tree & AED/QI2 | 4 | T1 | T1 | 0.29 | 0.66 | X |  | X | X | X |  |
| Zm00001d050265 | Gene Tree & AED/QI2 | 3 | T2 | T3 | 0.29 | 0.75 |  |  | X | X |  | X |
| Zm00001d049995 | Gene Tree & AED/QI2 | 1 | T1 | T1 | 0.29 | 0.75 |  |  |  | X |  | X |
| Zm00001d020971 | Gene Tree & AED/QI2 | 2 | T2 | T2 | 0.31 | 0.75 |  | X | X | X |  |  |
| Zm00001d026148 | Gene Tree & AED/QI2 | 2 | T1 | T2 | 0.39 | 0.5 | X | X |  | X |  | X |
| Zm00001d042445 | AED/QI2 | 1 | T1 | T1 | 0.02 | 0.4 |  |  |  |  |  | X |
| Zm00001d036242 | AED/QI2 | 1 | T1 | T1 | 0.02 | 0.66 |  |  |  |  |  | X |
| Zm00001d025355 | AED/QI2 | 1 | T1 | T1 | 0.04 | 0.75 | X |  |  |  |  |  |
| Zm00001d011277 | AED/QI2 | 1 | T1 | T1 | 0.09 | 0.33 |  |  | X | X |  |  |
| Zm00001d019030 | AED/QI2 | 1 | T1 | T1 | 0.13 | 0.5 | X | X |  |  |  |  |
| Zm00001d032969 | AED/QI2 | 1 | T1 | T1 | 0.18 | 0.5 |  | X |  |  |  |  |
| Zm00001d013777 | AED/QI2 | 1 | T1 | T1 | 0.27 | 0.42 |  | X |  |  |  |  |
| Zm00001d005890 | AED/QI2 | 1 | T1 | T1 | 0.29 | 0.5 |  |  |  |  |  | X |
| Zm00001d048702 | AED/QI2 | 2 | T1 | T1 | 0.09 | 0.5 |  | X | X | X | X |  |
| Zm00001d017111 | AED/QI2 | 2 | T1 | T2 | 0.11 | 0.5 | X |  |  | X | X |  |
| Zm00001d018415 | AED/QI2 | 2 | T2 | T1 | 0.11 | 0.66 | X |  |  |  | X |  |
| Zm00001d001960 | AED/QI2 | 2 | T1 | T2 | 0.16 | 0.5 |  |  |  | X |  | X |
| Zm00001d017288 | AED/QI2 | 2 | T1 | T2 | 0.17 | 0.66 | X |  |  | X | X |  |
| Zm00001d028963 | AED/QI2 | 2 | T1 | T2 | 0.18 | 0.6 |  | X |  | X | X |  |
| Zm00001d034635 | AED/QI2 | 2 | T1 | T2 | 0.19 | 0.66 | X |  |  |  | X |  |
| Zm00001d002982 | AED/QI2 | 2 | T1 | T1/T2 | 0.22/  0.23 | 0.5/  0.33 | X |  |  |  | X |  |
| Zm00001d015366 | AED/QI2 | 2 | T1 | T2 | 0.27 | 0.75 |  |  |  | X | X |  |
| Zm00001d003913 | AED/QI2 | 2 | T1 | T1 | 0.28 | 0.75 | X |  |  | X |  |  |
| Zm00001d002449 | AED/QI2 | 2 | T1 | T2 | 0.3 | 0.5 |  |  |  | X | X |  |
| Zm00001d049610 | AED/QI2 | 2 | T1 | T2 | 0.33 | 0.66 | X |  |  | X | X |  |
| Zm00001d042287 | AED/QI2 | 2 | T1 | T2 | 0.39 | 0.5 | X |  |  | X | X |  |
| Zm00001d021526 | AED/QI2 | 2 | T1 | T2 | 0.39 | 0.66 |  |  |  | X | X |  |
| Zm00001d041882 | AED/QI2 | 3 | T2 | T2 | 0.12 | 0.33 | X | X |  |  | X |  |
| Zm00001d024009 | AED/QI2 | 3 | T1 | T2 | 0.12 | 0.7 |  |  |  | X | X |  |
| Zm00001d014947 | AED/QI2 | 3 | T1 | T3 | 0.19 | 0.5 | X | X |  | X | X |  |
| Zm00001d015618 | AED/QI2 | 3 | T1 | T2 | 0.21 | 0.66 | X |  |  | X | X |  |
| Zm00001d023904 | AED/QI2 | 3 | T1 | T1 | 0.24 | 0.75 |  |  |  | X | X |  |
| Zm00001d003157 | AED/QI2 | 3 | T1 | T3 | 0.25 | 0.5 |  |  |  | X | X |  |
| Zm00001d019565 | AED/QI2 | 3 | T1 | T2/T3 | 0.27/  0.28 | 0.5 |  |  |  | X | X |  |
| Zm00001d012561 | AED/QI2 | 3 | T1 | T3 | 0.3 | 0.5 | X |  |  |  | X |  |
| Zm00001d025267 | AED/QI2 | 3 | T2 | T2 | 0.35 | 0.5 | X |  |  |  | X |  |
| Zm00001d043175 | AED/QI2 | 3 | T1 | T3 | 0.41 | 0.5 |  |  |  | X | X |  |
| Zm00001d049239 | AED/QI2 | 4 | T1 | T2 | 0.29 | 0.5 |  | X |  | X | X |  |
| Zm00001d045735 | AED/QI2 | 4 | T2 | T2 | 0.23 | 0.75 |  | X |  |  | X |  |
| Zm00001d050032 | AED/QI2 | 4 | T3 | T2 | 0.25 | 0.72 | X | X |  |  |  |  |
| Zm00001d039260 | AED/QI2 | 4 | T2 | T4 | 0.4 | 0.66 | X |  |  | X | X |  |
| Zm00001d014858 | AED/QI2 | 4 | T1 | T2 | 0.37 | 0.66 | X |  |  | X | X |  |
| Zm00001d013631 | AED/QI2 | 4 | T1 | T1 | 0.38 | 0.6 | X | X |  |  | X |  |
| Zm00001d020636 | AED/QI2 | 4 | T1 | T4 | 0.43 | 0.75 | X |  |  | X | X |  |
| Zm00001d050350 | AED/QI2 | 1 | T1 | T1 | 0.17 | 0.75 | X |  |  | X |  |  |
| Zm00001d044705 | Gene Triage | 1 | T1 | - | 0.05 | 1 |  |  |  | X |  | X |
| Zm00001d052537 | Gene Triage | 1 | T1 | - | 0.05 | 1 |  |  |  |  |  | X |
| Zm00001d014842 | Gene Triage | 1 | T1 | - | 0.06 | 0 |  | X |  |  |  | X |
| Zm00001d020383 | Gene Triage | 1 | T1 | - | 0.06 | 1 |  |  |  | X |  |  |
| Zm00001d040331 | Gene Triage | 1 | T1 | - | 0.07 | 1 |  |  |  |  |  | X |
| Zm00001d003006 | Gene Triage | 2 | T1 | - | 0.07 | 1 |  |  |  |  |  | X |
| Zm00001d036370 | Gene Triage | 1 | T1 | - | 0.08 | 0 |  | X |  |  |  | X |
| Zm00001d037737 | Gene Triage | 3 | T1 | - | 0.1 | 1 | X |  |  | X | X |  |
| Zm00001d032922 | Gene Triage | 1 | T1 | - | 0.1 | 1 |  |  |  |  |  | X |
| Zm00001d045054 | Gene Triage | 1 | T1 | - | 0.11 | 0.8 | X |  |  | X |  |  |
| Zm00001d013258 | Gene Triage | 1 | T1 | - | 0.11 | 1 | X |  |  | X |  | X |
| Zm00001d045055 | Gene Triage | 1 | T1 | - | 0.13 | -1 | X |  |  | X |  |  |
| Zm00001d037439 | Gene Triage | 1 | T1 | - | 0.13 | 1 |  | X |  |  |  |  |
| Zm00001d019648 | Gene Triage | 2 | T2 | - | 0.14 | 1 |  |  |  | X |  | X |
| Zm00001d042879 | Gene Triage | 4 | T2 | - | 0.16 | 1 |  | X |  | X | X | X |
| Zm00001d002353 | Gene Triage | 1 | T1 | - | 0.17 | 0 |  |  |  | X |  | X |
| Zm00001d047522 | Gene Triage | 4 | T2 | - | 0.17 | 0.8 | X |  |  | X | X |  |
| Zm00001d024698 | Gene Triage | 3 | T2 | - | 0.18 | 1 | X |  |  |  | X | X |
| Zm00001d003804 | Gene Triage | 1 | T1 | - | 0.18 | 1 |  |  |  |  |  | X |
| Zm00001d017614 | Gene Triage | 4 | T3 | - | 0.21 | 0.77 |  |  |  | X | X |  |
| Zm00001d009431 | Gene Triage | 3 | T1 | - | 0.21 | 0.9 |  |  |  |  |  | X |
| Zm00001d019148 | Gene Triage | 4 | T2 | - | 0.21 | 1 |  |  |  | X | X |  |
| Zm00001d043146 | Gene Triage | 2 | T2 | - | 0.24 | 1 |  |  |  | X |  |  |
| Zm00001d038725 | Gene Triage | 1 | T1 | - | 0.25 | 1 |  |  |  | X |  |  |
| Zm00001d015450 | Gene Triage | 3 | T1 | - | 0.28 | 1 | X |  |  | X | X | X |
| Zm00001d041781 | Gene Triage | 4 | T1 | - | 0.28 | 1 |  | X |  | X | X |  |
| Zm00001d051465 | Gene Triage | 3 | T2 | - | 0.3 | 1 |  |  |  | X | X | X |
| Zm00001d006116 | Gene Triage | 1 | T1 | - | 0.31 | 0.83 | X | X | X | X |  |  |
| Zm00001d051898 | Gene Triage | 1 | T1 | - | 0.35 | 1 | X |  |  |  | X |  |
| Zm00001d018535 | Gene Triage | 3 | T3 | - | 0.37 | 1 |  | X |  |  |  |  |
| Zm00001d032249 | Gene Triage | 2 | T2 | - | 0.51 | 0.6 |  |  |  | X |  | X |
| Zm00001d039132 | Gene Triage | 1 | T1 | - | 0.6 | 0 | X | X | X | X | X | X |
| Zm00001d035760 | Gene Triage | 1 | T1 | - | 0.64 | -1 |  |  |  |  |  | X |
| Zm00001d037386 | Gene Triage | 1 | T1 | - | 0.01 | 1 |  |  |  | X | X |  |
