# Supplementary material for: Double triage to identify poorly annotated genes in maize: The missing link in community curation
Source: PLoS One. 2019 Oct 28;14(10):e0224086. doi: 10.1371/journal.pone.0224086 (PMC6816542; doi:10.1371/journal.pone.0224086)
Supplement: S3 Table — (DOCX) [file pone.0224086.s004.docx]

**S3 Table. Evaluation of invertases in maize**

| **Gene ID** | **Gene name** | **Transcript count** | **Error detection**  **method** | **Error flagged in trees** | **Canonical flagged by quality metrics** | **Main improvements** |
| --- | --- | --- | --- | --- | --- | --- |
| Zm00001d002830 | INVVR1 | 1 | not flagged | - | no | 9-nt exon (present in V3) |
| Zm00001d014947 | INVVR2 | 3 | AED/QI2 | - | no | - |
| Zm00001d054075 | INVVR3 | 2 | Gene Tree & AED/QI2 | exon loss | yes | extended exons 1-2 (non-canonical junctions) |
| Zm00001d025943 | pseudogene/novel | 1 | not flagged | - | no | 9-nt exon (OUR FINDING) |
| Zm00001d016708 | INVCW1 | 2 | not flagged | - | no | 9-nt exon (present in V3) |
| Zm00001d003776^a^ | INVCW2 | 1 | Gene Tree | 5'-loss | no | 9-nt exon (present in V3); not originally flagged by curators (GT false negative*****) |
| Zm00001d025355 | INVCW3 | 1 | AED/QI2 | - | yes | 9-nt exon (present in V3) |
| Zm00001d001941 | INVCW4 | 1 | Gene Tree | exon loss | no | TWO mini-exons: 9-nt & 19-nt (OUR FINDING). Also extended longest exon (wrong in V3) |
| Zm00001d025354 | INVCW5 | 1 | Gene Tree | exon gain | no | 9-nt exon (OUR FINDING) |
| Zm00001d001944 | INVCW6 | 1 | Gene Tree & AED/QI2 | exon gain | yes | 9-nt exon (OUR FINDING) |
| Zm00001d001943 | INVCW7 | 1 | not flagged | - | no | 9-nt exon (present in V3) |
| Zm00001d041991 | INVCW8 | 1 | Gene Tree & AED/QI2 | exon gain | yes | 9-nt exon (present in V3) |

**Legend.^. a^**Annotation errors for **Zm00001d003776** (INVCW2) were not originally detected with the gene tree visualizer. Therefore, the gene is not found in the “improved classical genes”, and was included in sensitivity calculations. Omission of the 9-nt exon resulted in this invertase being truncated at its 5’-end.
